# Acute feeding with almonds compared to a carbohydrate-based snack improves appetite-regulating hormones with no effect on self-reported appetite sensations: a randomised controlled trial

**DOI:** 10.1007/s00394-022-03027-2

**Published:** 2022-10-28

**Authors:** Sharayah Carter, Alison M. Hill, Jonathan D. Buckley, Sze-Yen Tan, Geraint B. Rogers, Alison M. Coates

**Affiliations:** 1 Alliance for Research in Exercise, Nutrition and Activity (ARENA), Allied Health & Human Performance, University of South Australia, GPO Box 2471, Adelaide, 5001 Australia; 2 Alliance for Research in Exercise, Nutrition and Activity (ARENA), Clinical and Health Sciences, University of South Australia, Adelaide, Australia; 3 School of Exercise and Nutrition Sciences, Institute for Physical Activity and Nutrition (IPAN), Deakin University, Geelong, Australia; 4 Microbiome Research, South Australian Health and Medical Research Institute (SAHMRI), Adelaide, Australia; 5 College of Medicine and Public Health, Flinders University, Bedford Park, Australia

**Keywords:** Nuts, Almonds, Appetite, Satiety, Gastrointestinal peptides

## Abstract

**Purpose:**

Early satiety has been identified as one of the mechanisms that may explain the beneficial effects of nuts for reducing obesity. This study compared postprandial changes in appetite-regulating hormones and self-reported appetite ratings after consuming almonds (AL, 15% of energy requirement) or an isocaloric carbohydrate-rich snack bar (SB).

**Methods:**

This is a sub-analysis of baseline assessments of a larger parallel-arm randomised controlled trial in overweight and obese (Body Mass Index 27.5–34.9 kg/m^2^) adults (25–65 years). After an overnight fast, 140 participants consumed a randomly allocated snack (AL [*n* = 68] or SB [*n* = 72]). Appetite-regulating hormones and self-reported appetite sensations, measured using visual analogue scales, were assessed immediately before snack food consumption, and at 30, 60, 90 and 120 min following snack consumption. A sub-set of participants (AL, *n* = 49; SB, *n* = 48) then consumed a meal challenge buffet ad libitum to assess subsequent energy intake. An additional appetite rating assessment was administered post buffet at 150 min.

**Results:**

Postprandial C-peptide area under the curve (AUC) response was 47% smaller with AL compared to SB (*p* < 0.001). Glucose-dependent insulinotropic polypeptide, glucagon and pancreatic polypeptide AUC responses were larger with AL compared to SB (18%, *p* = 0.005; 39% *p* < 0.001; 45% *p* < 0.001 respectively). Cholecystokinin, ghrelin, glucagon-like peptide-1, leptin and polypeptide YY AUCs were not different between groups. Self-reported appetite ratings and energy intake following the buffet did not differ between groups.

**Conclusion:**

More favourable appetite-regulating hormone responses to AL did not translate into better self-reported appetite or reduced short-term energy consumption. Future studies should investigate implications for longer term appetite regulation.

**ANZCTR Reference Number:**

ACTRN12618001861246 2018.

**Supplementary Information:**

The online version contains supplementary material available at 10.1007/s00394-022-03027-2.

## Introduction

The high prevalence of overweight and obesity is a major public health concern [1]. Obesity is characterised by an excess of body fat that impairs both physical and psychosocial health and well-being [2]. Long-term regulation of body weight is controlled by balancing energy intake with energy expenditure [3]. Understanding the role of specific food items and their impact on energy intake may assist in promoting weight reduction and weight loss maintenance for people with obesity [1].

Epidemiological studies have provided evidence that regular consumption of nuts may reduce the risk of obesity [4–7]. Although nuts are energy-dense, incorporating them into the diet has not been shown to increase body weight [8–16]. A recent meta-analysis reported no increase in body weight with diets that included nuts compared to nut-free diets, but did report reductions in waist circumference with consumption of almonds [17]. In another recent meta-analysis, a higher intake of nuts was associated with reductions in body weight and body fat [16].

It has been suggested that humans compensate for the energy from nuts by reducing intake of other foods at subsequent eating occasions [18]. This may be due to the satiating effects of nuts, which possibly results from their high protein, fibre, and unsaturated fatty acid content, in conjunction with their low glycaemic load [19–21]. Additionally, nuts are associated with higher postprandial thermogenesis, which may raise resting energy expenditure with long-term consumption and help to balance the energy from nuts [9, 22]. Finally, it has been suggested that the available energy from nuts is less than predicted by the Atwater factor due to incomplete lipid release for absorption, therefore, contributing less energy than expected [19, 23].

Adaptive responses resulting from nut consumption may reflect effects on hormones involved in appetite control [24]. Recent studies have suggested that nut consumption may influence appetite through the modulation of gastrointestinal and pancreatic peptides including glucagon-like peptide-1 (GLP-1) [13, 25, 26], glucose-dependent insulinotropic polypeptide (GIP) [25, 26], ghrelin [25, 27], peptide YY (PYY) and pancreatic polypeptide (PP) [26]. However, not all studies have reported the beneficial effects of nut consumption on appetite-regulating hormones [28–31], possibly reflecting the complexity of adaptive responses and differing study designs.

The purpose of this study was to compare the effects of eating almonds or a carbohydrate-based snack on appetite-regulating hormones, self-reported appetite ratings, and short-term energy intake. We hypothesised that almonds would have favourable effects on appetite-regulating hormones and self-reported appetite ratings, reducing subsequent energy intake compared to the carbohydrate-based snack, and thus providing insight into the association of nut consumption with a reduced risk of obesity.

## Materials and methods

### Ethics approvals and trial registration

Ethics approval was obtained from the University of South Australia Human Research Ethics Committee (201,436) and the trial was registered with the Australian and New Zealand Clinical Trials Registry (ATCRN12618001861246).

### Study setting, design and participants

Data reported here were obtained from a parallel-arm randomised controlled trial that was conducted between January 15, 2019 and March 10, 2021 in the research facilities of the Alliance for Research in Exercise, Nutrition and Activity Centre (ARENA) at the University of South Australia, Adelaide. Written informed consent was obtained from participants prior to participation. The intervention trial examined whether the inclusion of almonds or carbohydrate-rich snacks in an otherwise nut-free energy-restricted diet would promote weight loss and protect against weight regain. Energy requirements were calculated using the Schofield equation and physical activity captured via the International Physical Activity Questionnaire [32]. Energy recommendations for weight loss were set at 30% less than requirements. Participants then incorporated 15% of their energy-restricted diet as unsalted whole, natural Californian almonds with skins or a carbohydrate-rich snack (oven-baked fruit cereal bar and rice crackers), 6 days/week for 9 months. The full protocol for the larger study has been published [33]. This paper reports on outcomes from acute baseline appetite testing using the above-mentioned snack foods (See supplementary Table 1 for macronutrient composition of test foods).

### Eligibility, randomisation and allocation

Participants were males and females, aged 25–65 years, weight stable, non-smokers, with a BMI of 27.5–34.9 kg/m^2^ who were recruited from the general public (full inclusion/exclusion criteria were published previously) [33]. Randomisation by minimisation was used to assign participants to either the almond (AL) or the snack bar (SB) treatment groups based on age, sex and BMI. An investigator not involved in study outcome assessments performed the treatment allocation.

### Appetite assessments

Participants attended the clinic following an overnight fast (> 10 h). Baseline blood samples were taken using an antecubital vein catheter, after which participants consumed their randomly allocated snack (almonds or a carbohydrate-based snack) with 200 mls of water within a 10-min period. Repeat blood sampling (via canulation) was performed every 30 min post snack for 2 h. Water (200 mls) was given at 60 min, and 100 mls at 90 min after snack consumption. Immediately after each blood draw, protease inhibitors (Sigma P2714 and Millipore DPP4-010) were added to the specimen tube. All samples were stored as serum at − 80 °C until assayed in duplicate. Appetite hormones ghrelin, GIP, GLP-1, leptin, PP, PYY as well as C-peptide and glucagon were assessed using a multiplex analysis system (LUMINEX MAGPIX, Millipore, Merck). CCK was assessed using ELISA (Ray Biotech). All samples for the same participant were run in the same assay.

Participants were asked to rate their subjective appetite sensations by answering four questions at the time of each blood draw. Each question was answered using a visual analogue scale (VAS); a 10 cm horizontal line anchored at both ends so that answers could be indicated on a continuous scale. VAS for appetite assessment has been shown to have good validity, reliability, and reproducibility [34]. The questions were: “How hungry do you feel?” with anchor values ranging from “I am not hungry at all” (scored as 0) to “I have never been more hungry” (scored as 10); “How satisfied do you feel?” with anchor values ranging from “I am completely empty” (scored as 0) to “I cannot eat another bite” (scored as 10); “How full do you feel?”, with anchor values ranging from “Not at all full” (scored as 0) to “Totally full” (scored as 10), and “How much do you think you could eat now?” with anchor values ranging from “Nothing at all” (scored as 0) to “A lot” (scored as 10). To avoid a participant’s response to each set of 4 VAS questions being biassed by their responses to the previous set, each paper set of 4 questions was taken from the participant before the next set was provided.

### Meal challenge buffet

In a sub-set of participants, a buffet meal was provided 2 h after test food consumption. The number of participants who consumed the buffet was limited due to the impact of COVID-19 lockdowns. Participants were given 30 min to eat as much or as little as they liked. The buffet was nut-free and provided a selection of core and discretionary foods and beverages, as defined by the Australia Dietary Guidelines, in generous volumes, and with limited predefined units (See supplementary Table 2 for a list of buffet foods). Food consumed (weighed before and after) was assessed for total energy via Foodworks Nutritional Analysis Software V.9 (Xyris Software, Brisbane, Queensland, Australia). A final set of VAS were performed immediately post buffet at 150 min.

### Statistics

All statistical analyses were performed using the SPSS for Windows V.24.0. (SPSS, Inc., Chicago, IL, USA). Sample size calculations were based on the primary outcome (change in weight) from the larger study and are detailed in the protocol paper [33]. Log transformation was performed on outcome variables when needed to improve normality, and the results were exponentiated for reporting purposes. Group characteristics at baseline were compared using mixed models analysis. Area under the curve (AUC) was calculated using the standard trapezoidal rule for appetite-regulating hormones and VAS appetite ratings. AUC was calculated only when full data sets were available. A linear mixed model analysis was used to compare AUC results by group. Age, sex and BMI were included in the models and effects of each reported. Appetite-regulating hormones and VAS appetite ratings were also assessed at each time point using mixed model analysis. Bonferroni’s test was used for multiple post hoc contrasts. Mixed model analyses were also used to assess total energy, core and discretionary food consumed at the buffet using the same covariates. All data are presented as means ± standard error (SE). The level of significance accepted was 0.05.

## Results

### Participant flow and baseline characteristics

**Fig. 1:**
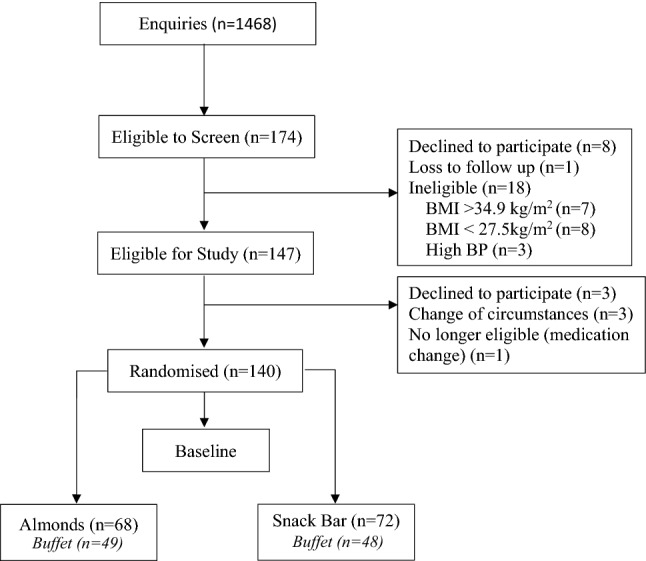
Consort flow diagram

A total of 140 participants completed the assessments (Male = 42, Female = 98, Age 47.5 ± 10.8 years, BMI 30.7 ± 2.3 kg/m^2^) and a sub-set of participants (AL, *n* = 49; SB, *n* = 48) completed the buffet. Figure 1 shows a flow diagram of participants who were screened and enrolled in the study. Participant characteristics and appetite assessment data (Table 1) were not significantly different between groups.

**Table 1:** Participant baseline characteristics

Characteristics	Almonds (*n* = 68)	Snack bar (*n* = 72)
Age, years	48 (1.3)	47 (1.3)
Sex–female (%)	48 (71)	50 (69)
Sex–male (%)	20 (29)	22 (31)
Weight, kg	87.8 (0.8)	87.7 (0.8)
BMI, m/kg^2^	30.7 (0.1)	30.7 (0.1)
Appetite-regulating hormones*		
CCK, pg/ml	281.9 (18.5)	271.0 (17.9)
C-peptide, ng/ml	1.1 (0.1)	1.1 (0.1)
Ghrelin, pg/ml	43.4 (6.7)	35.9 (6.5)
GIP, pg/ml	46.9 (9.2)	44.4 (8.8)
GLP-1, pg/ml	22.8 (8.4)	10.0 (8.1)
Glucagon, pg/ml	50.8 (4.5)	44.9 (4.4)
Leptin, ng/ml	16.5 (1.1)	15.5 (1.0)
PP, pg/ml	69.1 (13.6)	65.3 (13.1)
PYY, pg/ml	54.1 (5.8)	50.4 (5.6)
Hunger/Satiety Measures		
VAS Hunger, cm	3.6 (0.4)	4.1 (0.4)
VAS Satisfaction, cm	3.7 (0.3)	3.4 (0.3)
VAS Fullness, cm	3.3 (0.3)	2.9 (0.3)
VAS Appetite, cm	5.5 (0.3)	5.6 (0.3)

All *p* values are > 0.05 for between group differences.*Appetite-regulating hormone data is complete for 112 participants (AL *n* = 54, SB *n* = 58). Abbreviations: body mass index *BMI*, Cholecystokinin *CCK*, glucose-dependent insulinotropic polypeptide *GIP*, glucagon-like peptide-1 *GLP-1*, pancreatic polypeptide *PP*, peptide *YY*, *PYY*, Visual Analogue Scale *VAS*

### Appetite-regulating hormones

**Fig. 2:**
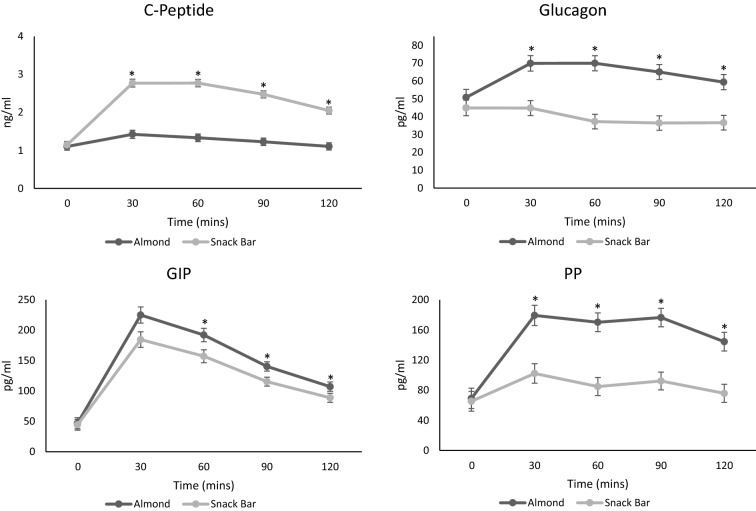
C-peptide, Glucagon, Glucose-dependent Insulinotropic Polypeptide (GIP) and Pancreatic Polypeptide (PP) Concentrations. Mean ± SE timepoint comparison * *p* =  < 0.05. Almond, *n* = 54; Snack Bar, *n* = 58.

C-peptide AUC response was significantly smaller in AL compared to SB (46.9%, *P* < 0.001) (Table 2). Timepoint comparisons indicated a lower C-peptide concentration at 30, 60, 90 and 120 min (*P* < 0.001 for all time points) in AL compared to SB (Fig. 2).

**Table 2:** Mean AUC for appetite hormones

AUC 0–120 min					
	Almonds (*n* = 54)		Snack bar (*n* = 58)		
Parameters	Mean	SE	Mean	SE	*P* values
CCK (pg/ml x min)	37,092	2113	34,730	2039	0.468
C-peptide (ng/ml x min)	153	10	288	10	< 0.001
Ghrelin (pg/ml x min)	5105	738	4214	712	0.207
GIP (pg/ml x min)	19,068	1122	15,672	1082	0.005
GLP-1 (pg/ml x min)	3562	979	1937	945	0.129
Glucagon (pg/ml x min)	7799	494	4780	476	< 0.001
Leptin (ng/ml x min)	1816	127	1698	123	0.328
PP (pg/ml x min)	18,952	1443	10,522	1392	< 0.001
PYY (pg/ml x min)	7504	666	6512	642	0.236

Cholecystokinin *CCK, *glucose-dependent insulinotropic polypeptide *GIP, *glucagon-like peptide-1 *GLP-1*, pancreatic polypeptide *PP*, peptide *YY*, *PYY*

The AL GIP AUC response was significantly larger than the response for SB (17.8%, *P* = 0.005) (Table 2). Higher concentrations occurred at time points 60 (*P* = 0.010), 90 (*P* = 0.003) and 120 min (*P* = 0.005) in AL compared to SB (Fig. 2).

The AL glucagon AUC response was significantly larger than the response for SB (38.7%, *P* < 0.001) (Table 2). Timepoint comparisons indicated a higher glucagon concentration at 30, 60, 90 and 120 min (*P* < 0.001 for all time points) in AL compared to SB (Fig. 2).

PP AUC response was significantly larger in AL compared to SB (44.5%, *P* < 0.001) (Table 2). Higher concentrations occurred at time point 30, 60, 90 and 120 min (*P* < 0.001 for all) in AL compared to SB (Fig. 2).

AUC for CCK, ghrelin, GLP-1, leptin and PYY did not differ between groups. Timepoint comparisons indicated a higher GLP-1 concentration at 60 (*P* = 0.015), 90 (*P* < 0.001) and 120 min (*P* = 0.024) in AL compared to SB (Supplementary Fig. 1).

### Subjective appetite ratings

**Fig. 3:**
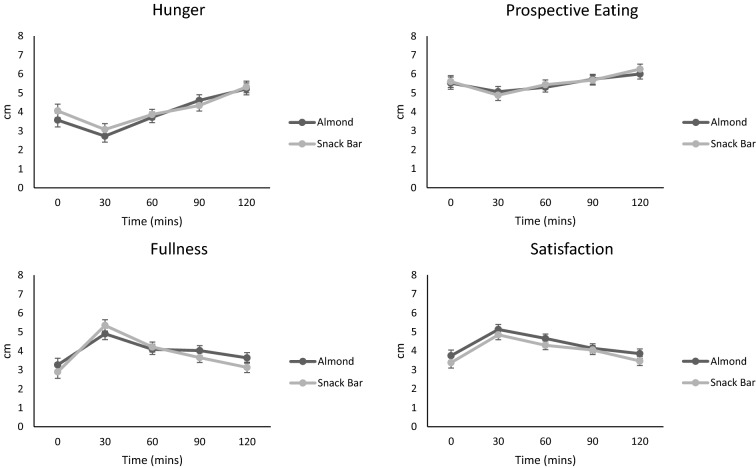
Self-reported appetite sensations measured using VAS. Mean ± SE timepoint comparison. Almond, *n* = 67; Snack Bar, *n* = 70

There was no evidence of a difference in self-reported appetite sensations (feelings of hunger, fullness, satisfaction and prospective food consumption [prospective eating]), obtained via VAS, to the different test snacks. In both groups, hunger and prospective eating decreased post snack and steadily increased over the remainder of the testing period. Similarly, fullness and satisfaction increased in both groups post snack and decreased over the remainder of the testing period (Fig. 3).

### Meal challenge buffet energy intake

**Fig. 4:**
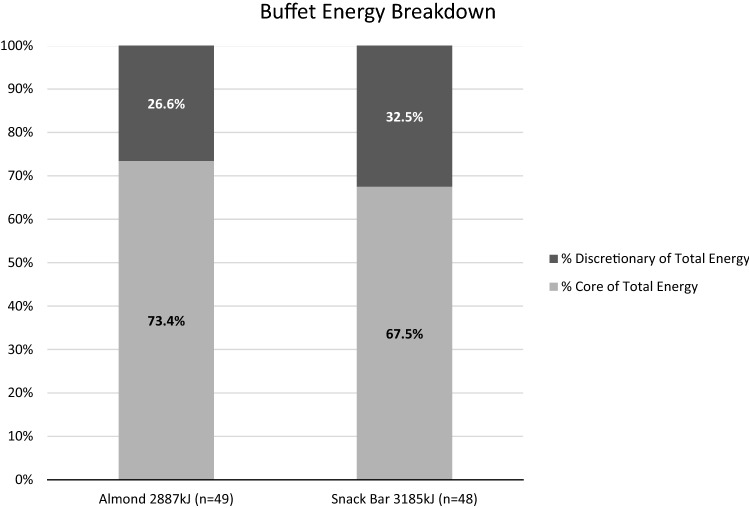
Proportion of total energy contributed by core and discretionary foods at buffet

There was no significant difference in total energy intake (AL 2887 [194] kJ, SB 3185 [196] kJ; *p* = 0.286) or energy from core (AL 2120 [118] kJ, SB 2150 [119] kJ; *p* = 0.860) or discretionary foods (AL 767 [132] kJ, SB 1035 [133] kJ; *p* = 0.158) between groups (Fig. 4). Males consumed more total energy (3682 [359] kJ) compared to females (2834 [145] kJ; *p* = 0.036) and more energy from core foods (males 2787 [240] kJ, females 1933 [78] kJ; *p* = 0.002). There was no sex difference in discretionary energy intake but there was an age difference, with younger people (based on median age 48 years) consuming more energy from discretionary foods (younger 1204 [159] kJ, older 624 [95] kJ; *p* = 0.003). Following the buffet meal (time point 150 min), the VAS responses for hunger, fullness, satisfaction, and prospective eating were not different between groups.

## Discussion

As rates of overweight and obesity continue to rise throughout the developed world, it is important to understand the mechanisms that regulate eating behaviours. Satiation and satiety are important factors in regulating food intake by suppressing hunger and hence food intake [35]. Foods that assist with appetite control help to promote energy balance and consequently assist with weight management [36].

Our study demonstrates that the consumption of almonds resulted in a smaller C-peptide response and a larger GIP, GLP-1 (timepoint comparisons only), glucagon and PP response compared to consuming an isocaloric carbohydrate-rich snack bar. Similar results have been reported in other studies investigating the effects of consuming nut (walnuts and pistachios) on appetite-regulating hormones [13, 25, 26]. Like other nut studies, we found no effect of almonds on CCK, ghrelin, leptin and PYY compared to the snack bar [26, 37]. In comparison, a crossover study in 7 men with type 2 diabetes compared a meal containing almonds and white bread with an isocaloric macronutrient-matched control meal (white bread, butter and cheese) and found the almond meal resulted in higher GLP-1 serum concentrations, decreased hunger and desire to eat, and increased fullness over 4 h [38]. The differences between our study and the work of Bodnaruc et al. [38] are likely due to the study by Bodnaruc et al. being conducted in men with type 2 diabetes whereas the present study was performed in health adults, but may also have been influenced by the different macronutrient composition of meals consumed, the difference in energy of test meals and the differences in the length of time that responses were measured after food consumption.

The nutritional profile of nuts, which includes high levels of protein, fibre, and unsaturated fatty acids, has been suggested to contribute to their satiating properties [19] and to confer a protective effect against development of cardiometabolic conditions [39]. C-peptide is a part of proinsulin that is cleaved before co-secretion with insulin from the β-cells of the pancreas [40]. The reduced C-peptide response seen in AL reflects lower insulin secretion compared to SB, which would have resulted from the lower carbohydrate content of AL compared with SB. A low carbohydrate content is a characteristic of nuts in general, not just almonds, and results in lower insulin and C-peptide responses following their consumption compared with carbohydrate-rich foods. This may have implications for development of both diabetes and cardiovascular disease due to improved insulin sensitivity [41]. Other studies have demonstrated that when nuts are added to a high carbohydrate food [25, 42], or a carbohydrate-rich meal [43], they reduce the glucose response and, over time, may aid in reducing insulin resistance.

Glucose-dependent insulinotropic polypeptide (GIP) is a pancreatic hormone with receptors in the β-cells of the pancreas which promote insulin secretion [44]. GIP activity is glucose-dependent, stimulating glucagon secretion when glucose concentrations are low [45]. Glucagon promotes satiety [46] and may promote weight loss via increased thermogenesis, energy expenditure, and fatty acid oxidation [46]. The increased GIP and glucagon response in the AL group is likely due to the low carbohydrate and high fat and protein content of almonds. In keeping with our findings, Kendall et al. reported greater increases in GIP in an acute crossover feeding trial with pistachios compared to white bread in adults with metabolic syndrome [25]. However, Rock et al. reported lower levels of GIP in an acute crossover trial using walnuts vs. cream cheese and suggested that the type of fat, unsaturated compared to saturated fat, may play a role, with saturated fat promoting a greater GIP response [26].

GLP-1 is secreted in the ileum in response to carbohydrate and fat [47]. In this study we see an initial spike in GLP-1 in the SB group, likely due to the carbohydrate content of the snack bar. A more sustained and significantly higher response is seen in the later stages of testing in the AL group, likely due to the fat content of almonds. GLP-1 slows stomach and gut motility, which may affect appetite [47].

Pancreatic polypeptide (PP) is secreted from endocrine cells in the pancreas in response to carbohydrate, protein and fat, with fat being the most potent stimulus and carbohydrate the least potent [48]. PP is also secreted in response to secretion of GIP [49]. The higher fat and protein content of AL compared to SB, as well as the greater increase in GIP in AL compared with SB, might therefore have contributed to the greater PP response in AL. PP is an anorectic hormone and acts on the hypothalamus to reduce food intake and has physiological effects, such as delayed gastric emptying, reducing appetite [50].

Although we observed significant effects of AL on C-peptide, GIP, GLP-1, glucagon and PP responses compared to SB, these responses alone without significant changes in other appetite-regulating hormones (e.g. CCK, PYY and ghrelin) may have been insufficient to trigger a meaningful appetite suppression. Therefore, it is not unexpected to see no differences in self-reported appetite sensations, or subsequent energy intake. However, appetite regulation is complex, and while the effects of the appetite hormones that we measured are well-characterised, there is not a direct correlation between appetite hormones, self-reported appetite ratings and subsequent energy intake within the literature [24, 51, 52]. In addition, the population evaluated in this study had BMIs in the overweight/obese range, and obesity is characterised by a resistance to appetite-regulating hormones, leading to a misalignment between physiological signals and the perceived satiety/satiation signal [53]. Interpreting the implications of changes in appetite hormone levels is complicated not only by factors such as the degree of adiposity, but also the form/composition of foods (e.g. portion size and sensory quality) and habitual meal patterns [24]. The volume of almonds (30-50 g) used in this study may have been too small to elicit subjective feeling of fullness. Neural feedback from stomach distension is linked to volume of food consumed and is important for triggering appetite hormone release (e.g. CCK and GLP-1) [47]. Hull et al. observed dose-dependent effects on hunger suppression and lower subsequent energy intake in a healthy weight population with higher doses of almonds (28 vs. 48 g) [54]. the low sensory quality, composition and timing of the snack may have impacted on sensations of satisfaction respective of usual meal patterns. Tan et al. reported an effect of meal timing, with a greater suppression of hunger when almonds were consumed as an afternoon snack compared to with breakfast, morning tea, or lunch [9]. Thus, despite differences in appetite-regulating hormones being observed between AL and SB, these other factors which can influence satiation and satiety might also have had an influence on subjective appetite resulting in no difference in energy intake at the buffet.

Although not significant, the AL group consumed 300 kJ less energy in the meal challenge than the SB group, 270 kJ of which came from discretionary foods, which may be a clinically important benefit in weight management. This occurred despite no significant difference in subjective appetite ratings. Ratings of appetite may reflect motivation or drive to eat, but the amount of food eaten may be influenced by factors other than motivation such as habit, expectations or availability [55].

### Strengths and limitations

This study was conducted prior to and during the COVID-19 pandemic, and restrictions on clinical research which occurred once the pandemic began prevented the study team from continuing the food buffet protocol. Thus, only those participants who completed the study prior to the onset of the pandemic completed the buffet meal component.

This study included participants with overweight or obesity, an important population group for appetite management. However, as individuals with elevated adiposity may respond differently, future research should examine differences between weight categories, specifically in a healthy weight population for the prevention of weight gain. While matching test foods for energy is important, future studies should also match on volume. The discrepancy between volume of almonds, half the gram amount of the snack bar, may have had an impact on results (See supplementary Table 1). Short-term, acute/early phase satiety responses were measured over a 2-h period. Brown et al. demonstrated that reduced energy intake occurred over 24 h and appetite effects may therefore need to be measured over a longer period [56]. In addition, future studies might consider standardising the evening meal and exercise the day before testing. Longer term appetite assessment is also needed to further understand the effects of nuts, appetite and weight management.

## Conclusion

Foods that promote satiety help to regulate energy balance and may assist with weight management. Future studies should consider test food dose and composition carefully as the volume of food, its sensory qualities, and the acceptance of the food respective of usual meal patterns, may be important in eliciting a feeling of fullness and satisfaction.

Appetite hormone responses may be skewed in obesity, so testing in a healthy weight population may provide additional insight into population-based differences. In addition, measures of glucose and insulin would be useful to further explore metabolic responses, and testing in populations with diabetes is warranted.

This study focuses on early phase satiety, so measuring responses over the long-term and after weight loss will better model the studies relating nut consumption to weight management.

## Supplementary Information

Below is the link to the electronic supplementary material.

Supplementary file1 (PDF 191 KB)
