# Supplementary material for: Acute feeding with almonds compared to a carbohydrate-based snack improves appetite-regulating hormones with no effect on self-reported appetite sensations: a randomised controlled trial
Source: Eur J Nutr. 2022 Oct 28;62(2):857–66. doi: 10.1007/s00394-022-03027-2 (PMC9614749; doi:10.1007/s00394-022-03027-2)
Supplement: Supplementary file 1 — Supplementary file1 (PDF 191 KB) [file 394_2022_3027_MOESM1_ESM.pdf]

Supplementary Table 1. Macronutrient Composition of Test Foods (per serve and per 100g)

	5000kJ Energy Restricted Diet		6300kJ Energy Restricted Diet		7600kJ Energy Restricted Diet		Per 100g	
	Almonds (30g)	Snack Bar (1.5 Bars/60g)	Almonds (40g)	Snack Bar (2 Bars/80g)	Almonds (50g)	Snack Bar (2.5 Bars/100g)	Almonds (100g)	Snack Bar (100g)
**Energy (kJ)**	716	762	954	1016	1193	1270	2385	1270
**Protein (g) (%)**	5.9 (14.0)	2.6 (5.9)	7.9 (14.0)	3.5 (5.9)	9.9 (14.0)	4.4 (5.9)	19.7 (14.0)	4.4 (5.9)
**Total fat (g) (%)**	15.2 (78.3)	0.6 (2.9)	20.2 (78.3)	0.8 (2.9)	25.3 (78.3)	1 (2.9)	50.5 (78.3)	1.0 (2.9)
**Saturated fat (g)**	1.1	0.2	1.5	0.2	1.9	0.3	3.8	0.3
**Polyunsaturated fat (g)**	3.9	0.2	5.1	0.3	6.4	0.4	12.8	0.3
**Monounsaturated fat (g)**	9.2	0.2	12.3	0.2	15.3	0.3	30.7	0.3
**Carbohydrate (g) (%)**	1.6 (3.6)	33.4 (72.4)	2.2 (3.6)	44.6 (72.4)	2.7 (3.6)	55.7 (72.4)	5.4 (3.6)	55.7 (72.4)
**Sugars (g)**	1.6	16.5	2.1	22	2.6	27.5	5.2	27.5
**Starch (g)**	0.1	16.9	0.1	22.6	0.1	28.2	0.2	28.2
**Fibre (g) (%)**	3.3 (3.7)	8.8 (18.8)	4.4 (3.7)	11.8 (18.8)	5.5 (3.7)	14.7 (18.8)	10.9 (3.7)	14.7 (18.8)

Almonds, Unsalted, Whole, Natural Almonds with Skin
Snack Bar, Weight Watchers Apple Crumble Bar
Foods analysed in Foodworks Nutritional Analysis Software version 9 (Xyris Software, Brisbane, QLD, Australia).

Supplementary Table 2. List of Buffet Foods

Food Type	Food Item
Bread	Helga’s Wholemeal Bread
Crisps/Chips	Smith’s Potato Chips (Original)
Chocolate	M&Ms
Plain Cracker	Arnott’s Cruskits (Original)
Dairy	Cheese Slices
Dairy	Cream Cheese
Dairy	Yoplait Vanilla Yoghurt
Dairy	Yoplait- Fruit Flavoured Yoghurt
Drink	Cottee’s Orange Cordial (Full Sugar)
Soft Drink/Soda	Kirk’s Lemon Squash (Full Sugar)
Soft Drink/Soda	Kirk’s Lemon Squash (Zero Sugar)
Drink	Water
Fruit	Sultanas
Fruit	Red Apple
Fruit (Tin)	Fruit Cups
Lollie/Mint	Mentos
Meat/Poultry/Fish	Tinned Tuna (with tomato and basil)
Meat/Poultry/Fish (Processed)	Ham
Meat/Poultry/Fish	Roast Chicken
Savory Cracker	Arnott’s BBQ Shapes
Spread	Vegemite
Spread	Jam
Spread	Polyunsaturated Margarine
Spread	Mayonnaise
Spread	Mustard
Sweet Biscuit	Arnott’s Tiny Teddy
Vegetable	Tomato

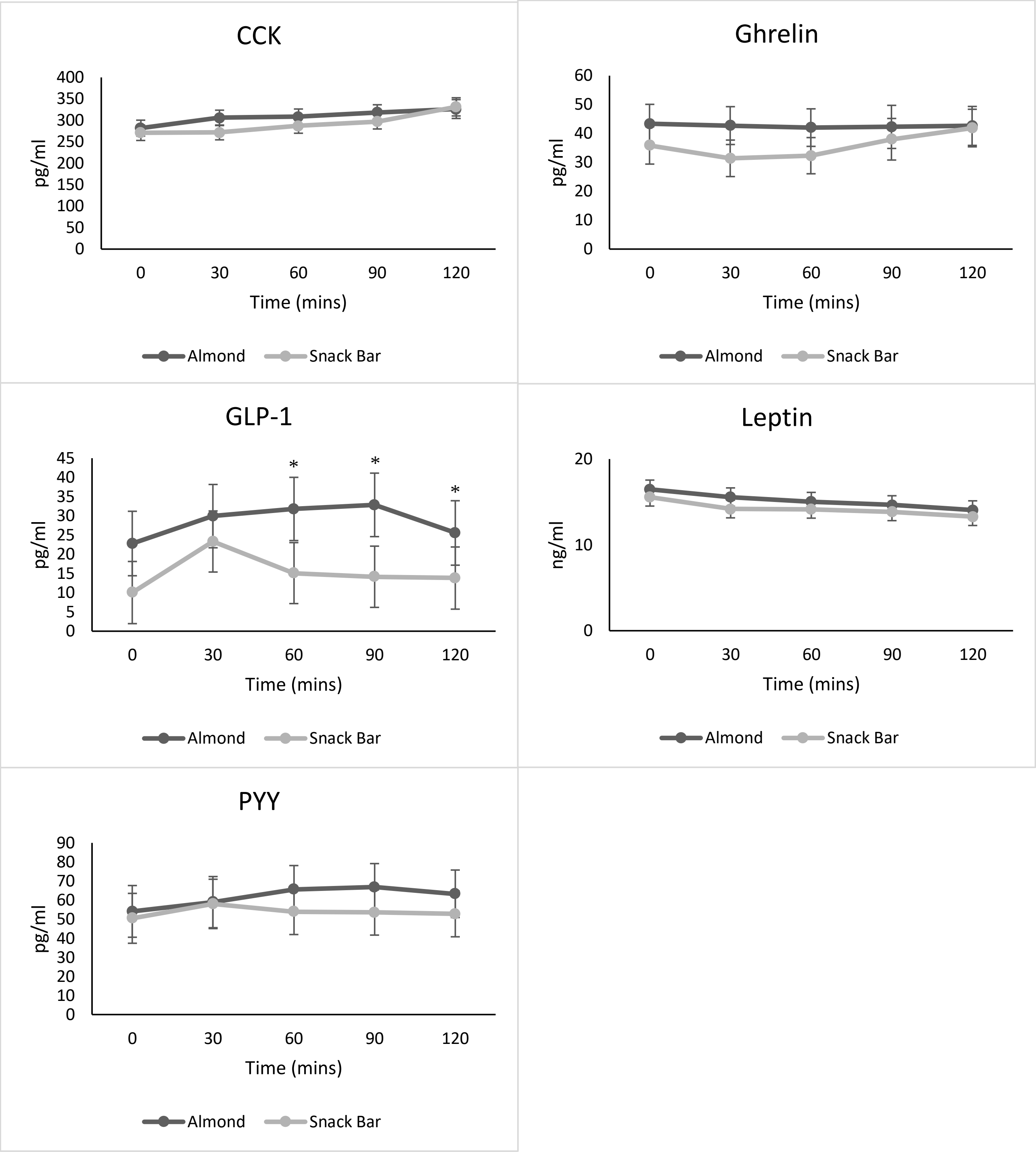

Supplementary Figure 1– Cholecystokinin (CCK), Ghrelin, Glucagon-like peptide-1 (GLP-1) Leptin, Peptide YY (PYY) Concentrations. Mean ± SE timepoint comparison * p=<0.05. Almond, n=54; Snack Bar, n=58.
